# A transcriptomic analysis in mice following a single dose of ibogaine identifies new potential therapeutic targets

**DOI:** 10.1038/s41398-024-02773-7

**Published:** 2024-01-19

**Authors:** Judit Biosca-Brull, Genis Ona, Lineth Alarcón-Franco, Maria Teresa Colomina

**Affiliations:** 1 Universitat Rovira i Virgili, Research Group in Neurobehavior and Health (NEUROLAB), Tarragona, Spain; 2 Universitat Rovira i Virgili, Department of Psychology and Research Center for Behavior Assessment (CRAMC), Tarragona, Spain; 3 Universitat Rovira i Virgili, Center of Environmental, Food and Toxicological Technology (TECNATOX), Reus, Spain; 4 ICEERS—International Center for Ethnobotanical Education, Research, and Services, Barcelona, Spain; 5 Universitat Rovira i Virgili, Department of Anthropology, Philosophy and Social Work, Tarragona, Spain; 6 Grupo de Investigación Infetarre, Facultad de Medicina, Universidad Cooperativa de Colombia, Medellín, Colombia

**Keywords:** Molecular neuroscience, Predictive markers

## Abstract

Ibogaine (IBO) is an atypical psychedelic with a complex mechanism of action. To date, the mechanisms that may underlie its anti-addictive effects are still not defined. This study aims to identify changes in gene expression induced by a single oral dose of IBO in the cortex of mice by means of a transcriptomic analysis for the first time. Our results showed significant alterations in gene expression in mouse frontal cortex samples 4 h after a single oral dose of IBO. Specifically, genes involved in hormonal pathways and synaptogenesis exhibited upregulation, while genes associated with apoptotic processes and endosomal transports showed downregulation. The findings were further corroborated through quantitative polymerase chain reaction (qPCR) analysis. However, the validation of gene expression related to hormonal pathways did not entirely align with the transcriptomic analysis results, possibly due to the brain region from which tissue was collected. Sex differences were observed, with female mice displaying more pronounced alterations in gene expression after IBO treatment. High variability was observed across individual animals. However, this study represents a significant advancement in comprehending IBO’s molecular actions. The findings highlight the influence of IBO on gene expression, particularly on hormonal pathways, synaptogenesis, apoptotic processes, and endosomal transports. The identification of sex differences underscores the importance of considering sex as a potential factor influencing IBO’s effects. Further research to assess different time points after IBO exposure is warranted.

## Introduction

Substance use disorders (SUDs) constitute one of the biggest challenges for treatment and recovery. It is estimated that about 0.5 million annual deaths worldwide are attributed to drug use [1]. Apart from fatal outcomes, several health problems related to SUDs can be observed over the short- and long-term, including intoxication, misuse, heart disease, depression, and more [2]. Treating SUDs generally has poor adherence and high rates of relapse [3, 4] which makes innovative approaches highly necessary.

Ibogaine (IBO) is an alkaloid naturally found in the root bark of some plants belonging to the Apocynaceae family, including *Tabernanthe iboga*. For decades, users and activists have claimed that IBO has remarkable anti-addictive properties [5]. However, research on this substance is scarce which is most likely due to its undesired effects, such as hallucinogenic effects [6] and cardiovascular toxicity [7]. There are no published trials supporting these claims, although the first Phase II trial was launched in 2020 by our group [NCT04003948], and the final results will be published soon.

The available evidence suggesting IBO is an efficacious treatment for SUDs is mainly based on preclinical and observational/open-label research. Various studies have shown that IBO decreases morphine, cocaine, alcohol, and nicotine self-administration (SA) in rats [8]. Three studies reported no reductions in conditioned place preference (CPP) with IBO when rats were trained in CPP using amphetamine and morphine [9–11]. It’s important to note that the CPP paradigm is typically utilized to evaluate Pavlovian conditioning, which involves automatic and involuntary responses. In contrast, SA tests encompass both Pavlovian and operant conditioning. The latter involves voluntary behaviors, and therefore, data from studies utilizing the SA paradigm are more translatable. In addition, it has been observed that IBO reduces naloxone-precipitated opioid withdrawal in rats [12–16]. Two reports [17, 18] indicate that naloxone-precipitated opioid withdrawal in rats remained unaffected by IBO, possibly due to the specific route of administration employed (subcutaneous). These findings suggest that the effects of IBO may have a central role influenced by first-pass metabolism. Indeed, O-demethylation through cytochrome P4502D6 (CYP2D6) converts IBO to noribogaine (NOR), its main metabolite, which has a higher volume of distribution and a longer half-life than the parent drug [19].

Observational research involving administering IBO to people with SUDs has shown promising results. For instance, Davis et al. [20] recruited a sample of people who underwent past IBO treatment and reported that 80% of them noted a drastic reduction in withdrawal symptoms. Fifty percent (50%) felt a reduction in opioid cravings and 30% did not use opioids again after the treatment. Other studies reported similar findings [21–24].

At least three open-label studies using IBO have been published [25–27]. Two of them highlighted enthusiastic results [26, 27]. However, they consist of a non-peer-reviewed chapter [27] and a commentary [26] mentioning non-published data with no specific details on methodology or reported outcomes. In addition, another publication by the same authors [28] references the commentary and claims there was no drug-related clinically relevant QT prolongation (which is data that is not reported anywhere). The researchers stated that the study population consisted of 191 patients, while the commentary reports 257 people. Due to these inconsistencies and the lack of published data, these results must be interpreted with caution. In fact, the third open-label trial [25] did not report such favorable results: 50% of patients reported QTcs above 500 ms and severe ataxia after IBO doses of 10 mg/kg.

The mechanisms through which IBO may exert its putative anti-addictive effects are still not fully understood. An early review summarized all the targets both IBO and NOR interact with [29]. However, more recent studies have suggested other potential targets/mechanisms [30–32], as well as sex-specific effects of IBO [33] which were hinted at in earlier preclinical studies [34]. A recent review collected all the available literature regarding the main targets of IBO/NOR in relation to the suggested benefits of SUD treatment [35]. These include affinity for both μ and κ opioid receptors, serotonin (SERT) and dopamine (DAT) transporters, N-methyl-d-aspartate (NMDA) and α3β4 nicotinic acetylcholine receptors, as well as increases in glial-derived (GDNF) and brain-derived (BDNF) neurotrophic factors, among others.

Due to the complex pattern of multi-target action, the scientific field would benefit from recently developed methodologies that provide a better understanding of the mechanisms of action of drugs, such as omics techniques. The entire molecular landscape affected by drugs can be revealed through these comprehensive techniques, instead of focusing on certain targets or receptors belonging to the G protein-coupled receptor (GPCR) family. This may identify previously unknown molecular players involved in drug response. For instance, some authors in the psychedelic research field claimed that the therapeutic effect of these substances can be attributable to modifications in the endocrine system [36]. This is because the hypothalamus possesses a notable concentration of 5-HT2A receptors, alongside other receptors implicated in the intricate workings of psychedelic substances. The administration of these drugs is correlated with the release of oxytocin and various other neuropeptides [37, 38] possibly modulating crucial aspects involved in psychopathology such as social cognition [39]. These potentially related—and still unexplored—mechanisms can be elucidated by exploring molecular changes in cells or tissues.

To date, few studies have introduced omics to the study of psychedelic drugs [40–44]. There are no published studies using these techniques with IBO. The aim of this study is to analyze the effects of a single IBO administration on gene expression in mice using transcriptomic analysis.

## Materials and methods

### Animals

Twelve eight-week-old C57BL/6J mice (six males and six females) were obtained from Charles River Laboratories (Barcelona, Spain). After one week of quarantine, male and female mice were assigned to the control (CNT) group or the IBO-treated group by simple randomization. The total number of animals for each group was 6, with three males and three females. Animals were maintained in a 12 h light/dark automatic cycle (the lights were on between 8 a.m. and 8 p.m.) with a controlled temperature (22 ± 2 °C) and humidity (50 ± 10%). Food (SAFE^®^ A04 diet, Panlab, Barcelona, Spain) and water were administered *ad libitum*. All the experiments of this study were conducted in compliance with the Spanish Royal Decree 53/2013 on the protection of animals used in experiments and the European Communities Council Directive (86/609/EC) and were approved by the Animal Care and Use Committee at the Rovira i Virgili University (Catalonia, Spain).

### Treatment and experimental design

Young mice were exposed to 60 mg/kg of IBO (12-Methoxyibogamine) provided by the International Center for Ethnobotanical Education, Research and Service (ICEERS) (Barcelona, Spain). The reported purity of IBO was 98.4% (±0.3%) as analyzed through Nuclear Magnetic Resonance (NMR) by Eurecat (Reus, Spain). IBO was dissolved in distilled water (the vehicle) and adjusted to administer the desired dose in 10 μL/g of body weight by gavage. The control group received the vehicle. The animals were exposed orally to mimic the administration of IBO in capsules in humans. In addition, the dose administered was between 40–80 mg/kg which is considered a medium dose and corresponds to the most frequent doses used in humans (10–25 mg/kg) corrected for body surface area [8].

In accordance with the results reported by Kubiliene et al. [45], the control and IBO-treated groups were euthanized by cervical dislocation 4 h after the oral administration, since this is the time when the peak concentration of IBO is observed in the brain. Brain samples were immediately removed, snap-frozen in liquid nitrogen, and stored at −80 °C until transcriptomic and gene expression analysis was done.

### Transcriptomic analysis

Frontal cortex tissue was selected to study changes in gene expression due to its involvement in different aspects related to drug dependence, such as reinforcement response to drugs during intoxication, activation during craving, and deactivation during withdrawal, as well as a generalized dysfunction in drug-dependent individuals [46]. Samples were sent to the Center for Omic Science (COS) (Reus, Spain) for RNA sequencing. RNA was extracted using the Purelink RNA mini kit from Invitrogen (Walthman, MA, USA) and quantified by a Qubit 2.0 Fluorometer (ThermoFisher Scientifics, Waltham, MA, USA). Then, the quality of the RNA was assessed using the Agilent TapeStation team and the Agilent RNA ScreeTape Assay (Agilent, Santa Clara, CA, USA). The sequencing libraries were created from 0.75 μg of RNA samples using the Illumina Stranded mRNA Prep (Illumina, San Diego, CA, USA) and quantified by microfluidic electrophoresis using Agilent’s TapeStation equipment and the Agilent DNA High Sensitivity ScreenTape kit (Agilent, Santa Clara, CA, USA). The length and concentration were determined in each sample. Finally, pools with a concentration of 750 pM were created. These pool sequencing libraries were done using NextSeq200 equipment from Illumina (San Diego, CA, USA).

The obtained gene database was screened for outliers which were then eliminated. Table 1 shows the total number of animals used.

**Table 1:** Animals used in this study.

Treatment	CNT		IBO	
Type of analysis	Omics	Gene expression	Omics	Gene expression
Males	3	3	3	3*
Females	2	3	3	3*

The asterisk indicates that one sample in *Oxt* and *Avp* genes was excluded because of expression values are more than 200.

### Gene expression analysis

The complementary RNA (cDNA) from frontal cortex tissues was synthesized from 1 mg of RNA samples using a Maxima First Strand cDNA Kit for RT-qPCR (ThermoFisher Scientific, Waltham, MA, USA). Then we performed the real-time polymerase chain reaction (qPCR) analysis with the Maxima SYBR Green/ROX qPCR Master Mix (2X) Kit (ThermoFisher Scientific, Waltham, MA, USA) and the Rotor-Gene Q Real-time Q cycler (Qiagen Inc., Hilden, Germany) to evaluate the gene expression of oxytocin (*Oxt*), arginine vasopressin (*Avp*), cerebellin 4 (*Cbln4*) and 2 (*Cbln2*) precursors and ubiquitin-specific peptidase 35 (*Usp35*). Duplicates of each RNA sample were included in the qPCR. We used the Rotor-Gene Q Real-Time PCR 2.0 software (Qiagen Inc., Hilden, Germany) to calculate the cycle threshold (Ct). Each sample was normalized to the housekeeping gene glyceraldehyde-3-phosphate dehydrogenase (*Gapdh*) (ΔCt) and standardized to the male control group (ΔΔCt) average to assess the relative gene expression levels in accordance with the 2-ΔΔCt method [47]. The primer sequences used for qPCR were as follows: *Oxt* (forward: 5′-TGGCTTACTGGCTCTGACCT-3′; reverse: 5′-GGCAGGTAGTTCTCCTCCTG-3′) [48], *Avp* (forward: 5′-CAGGATGCTCAACACTACGC-3′; reverse: 5′-CAGAATCCACGGACTCCCG-3′) [48], *Cbln4* (forward: 5′-GCACCGAGGAAAGGAATCTA-3′; reverse: 5′-TGCAGAGATGACTGGTTTTCC-3′) [49], *Cbln2* (forward: 5′-TGACCCTCAGATGGATTGCAC-3′; reverse: 5′-CTGCTGGGCTCTTGCTTTAAGC-3′) [50], *Usp35* (forward: 5′-TGCCATTAGCAGGATGATTGA-3′; reverse: 5′-AGCGAAACCTCGATCAAGATG-3′) [51] and the reference gene *Gapdh* (forward: 5′-ACAACTTTGGCATTGTGGAA-3′; reverse: 5′-AGCGAAACCTCGATCAAGATG-3′) [52].

### Statistical analysis

The sample size was calculated according to pharmacokinetic studies [45]. The data obtained was mapped against a reference genome using the alignment program HISAT2 2.2.1, while the annotation and quantification of the aligned reads were performed using StringTie 2.1.4. To investigate the changes in gene expression profiles induced by IBO treatment, we utilized R 4.3.0 and its specific package DESeq 1.40.1 to calculate the fold change (FC) values of each gene relative to the CNT group. Statistical analysis excluded genes that have less than five counts in each treated sample. The threshold for identifying significant differences was set at *p* adj. < 0.05. In addition, we calculated the effect size using Cohen’s *d*. Values above 0.8 indicate a large effect, values between 0.50 and 0.79 indicate a medium effect and values between 0.21 and 0.49 indicate small effects, while values below 0.20 indicate no effect [53].

Gene expression analysis was performed using SPSS 28.0 software (IBM Corp. Chicago, IL, USA). The homogeneity of variance was evaluated by the Levene test. A two-way analysis of variance (ANOVA) was conducted to assess significant differences between sex or treatment and their interactions. All the data are presented as the mean ± S.E.M, and statistical significance was set at *p* < 0.05.

## Results

### Screening for gene expression alterations

The first analysis compared the CNT and IBO-treated groups, regardless of sex. Table 2 demonstrates that in the total number of evaluated genes, seven were differentially expressed. Specifically, four genes showed a significant increase in expression after IBO administration (oxytocin (*Oxt*), vasopressin (*Avp*), cerebellin (*Cbln*) 2 and 4 precursors). Ubiquitin-specific peptidase 35 adaptor (*Usp35*), adaptor-related protein complex 5, beta 1 subunit (*Ap5b1*), and the predicted gene *Gm34306* showed a significant decrease (Table 2). Cohen’s d showed a large effect in all significant genes, except for *Cbln4* and *Usp35* which showed a medium effect, and *Cbln2* with a small effect.

**Table 2:**
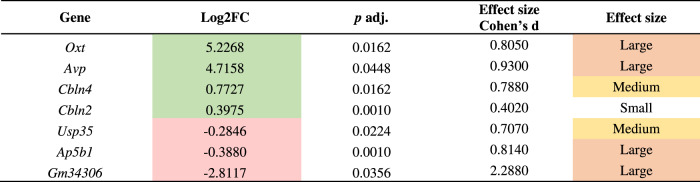
DESeq2 results of differentially expressed genes comparing CNT and IBO-treated groups.

Green log2FC indicates significant upregulated genes, while red log2FC indicates significant downregulated genes at *p* adj. < 0.05. Orange and yellow effect sizes indicate large and medium effects, respectively, whereas no color indicates a small effect according to Cohen’s *d*.

*FC* fold change.

The differences in gene expression changed when we evaluated males and females separately. In males, eight of the total number of evaluated genes were differentially expressed (Table 3), whereas there were 28 genes that showed expression alterations in females (Table 4). Male mice treated with IBO presented an upregulation of *Gm51898*, *Cbln4*, and interleukin 1 (IL1) receptor antagonists (*Il1rn*). Conversely, two predicted genes (*Gm36884* and *Gm6334)* were downregulated, as well as phospholipase A2 (*Pla2g4b*), and one of their inhibitors (*Pinlyp*) (Table 3). All the genes showed Cohen’s *d* values greater than 0.8, indicating a large effect.

**Table 3:**
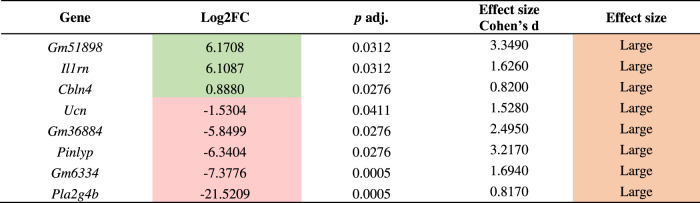
DESeq2 results of differentially expressed genes comparing male CNT and IBO-treated groups.

Green log2FC indicates significant upregulated genes, while red log2FC indicates significant downregulated genes at *p* adj. < 0.05. Orange effect size indicates a large effect according to Cohen’s *d*.

*FC* fold change.

**Table 4:**
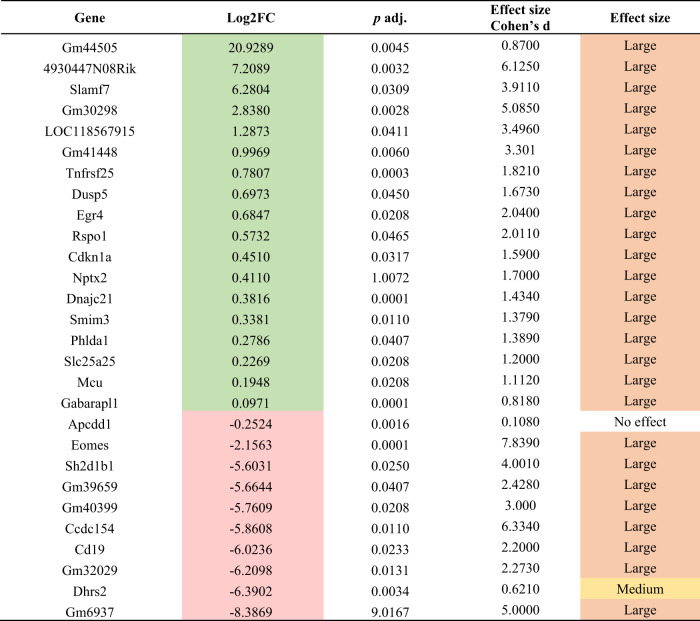
DESeq2 results of differentially expressed genes comparing female CNT and IBO-treated groups.

Green log2FC indicates significant upregulated genes, while red log2FC indicates significant downregulated genes at *p* adj. < 0.05. Orange and yellow effect sizes indicate large and medium effect, respectively, whereas no color indicates no effect according to Cohen’s *d*.

*FC* fold change.

Out of the 28 genes analyzed in female mice, 18 were found to be upregulated and 10 were downregulated. In particular, female mice treated with IBO showed an increase in the expression of neuronal pentraxin 2 (*Nptx2*), gamma-aminobutyric acid receptor-associated protein (*Gabarap11*), tumor necrosis factor receptor superfamily member 25 (*Tnfrsf25*), cyclin-dependent kinase inhibitor 1 A (*Cdkn1a*), pleckstrin homology like domain family A member 1 (*Phlda1*), early growth response 4 (*Egr4*), solute carrier family 25 member 25 (*Slc25a25*), mitochondrial calcium uniporter (*Mcu*), small integral membrane protein 3 (Smim3), SLAM family member 7 (*Slamf7*), dual specificity phosphatase 5 (*Dusp5*), DNAJ heat shock protein family (*Hsp40*) member C21 (*Dnajc21*), and R-spondin1 (*Rspo1*). Other genes such as tumor suppressor coiled-coil domain containing 154 (*Ccdc154*), the dehydrogenase/reductase 2 (*Dhrs2*), SH2 domain containing 1B1 (*Sh2d1b1*), eomesodermin (*Eomes*), CD19 antigen (*Cd19*) and adenomatous polyposis coli (*Apcdd1*) were decreased. In addition, we found uncharacterized and predicted genes that were also differentially expressed in IBO-treated females. The genes that showed overexpression were *LOC118567915, 4930447N08Rik, Gm44505, Gm30298*, and *Gm41448. Gm39659, Gm40399, Gm32029*, and *Gm6937* were downregulated (Table 4). Cohen’s *d* values were greater than 0.8 in all significant genes, except for *Dhrs2* and *Apcdd1* which showed medium and small effects, respectively.

### Validation of gene expression alterations

Based on the observed alterations in gene expression after 4 h of IBO administration, we aimed to validate the obtained results by comparing the CNT and IBO-treated groups using qPCR. For this purpose, we assessed the gene expression listed in Table 2, excluding Ap5b1 and the predicted gene Gm4306.

**Fig. 1:**
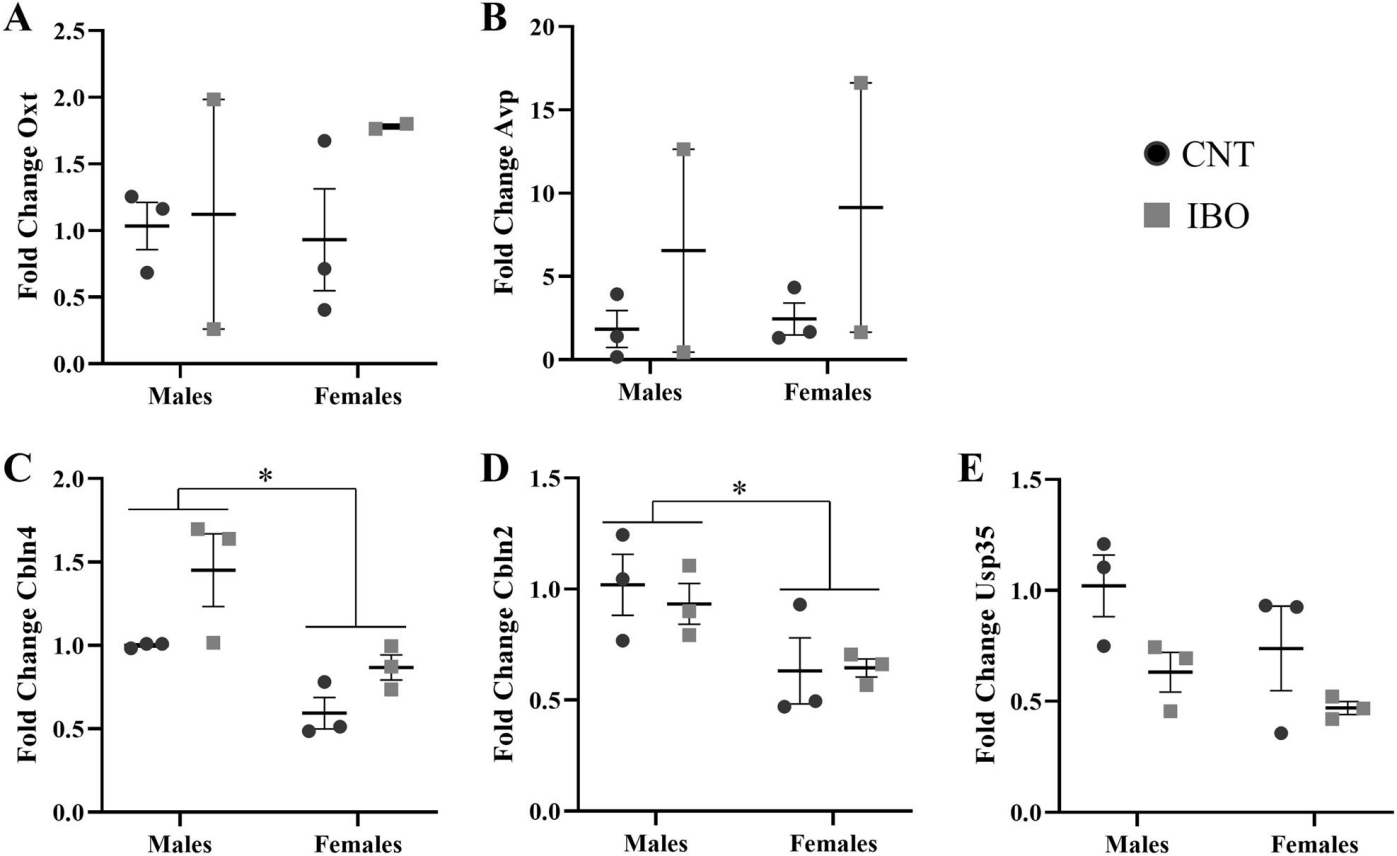
Frontal cortex gene expression determined by qPCR. *Oxt* (**A**), *Avp* (**B**), *Cbln4* (**C**), *Cbln2* (**D**) and *Usp35* (**E**). The symbol * indicates differences between sexes at *p* < 0.05.

**Fig. 2:**
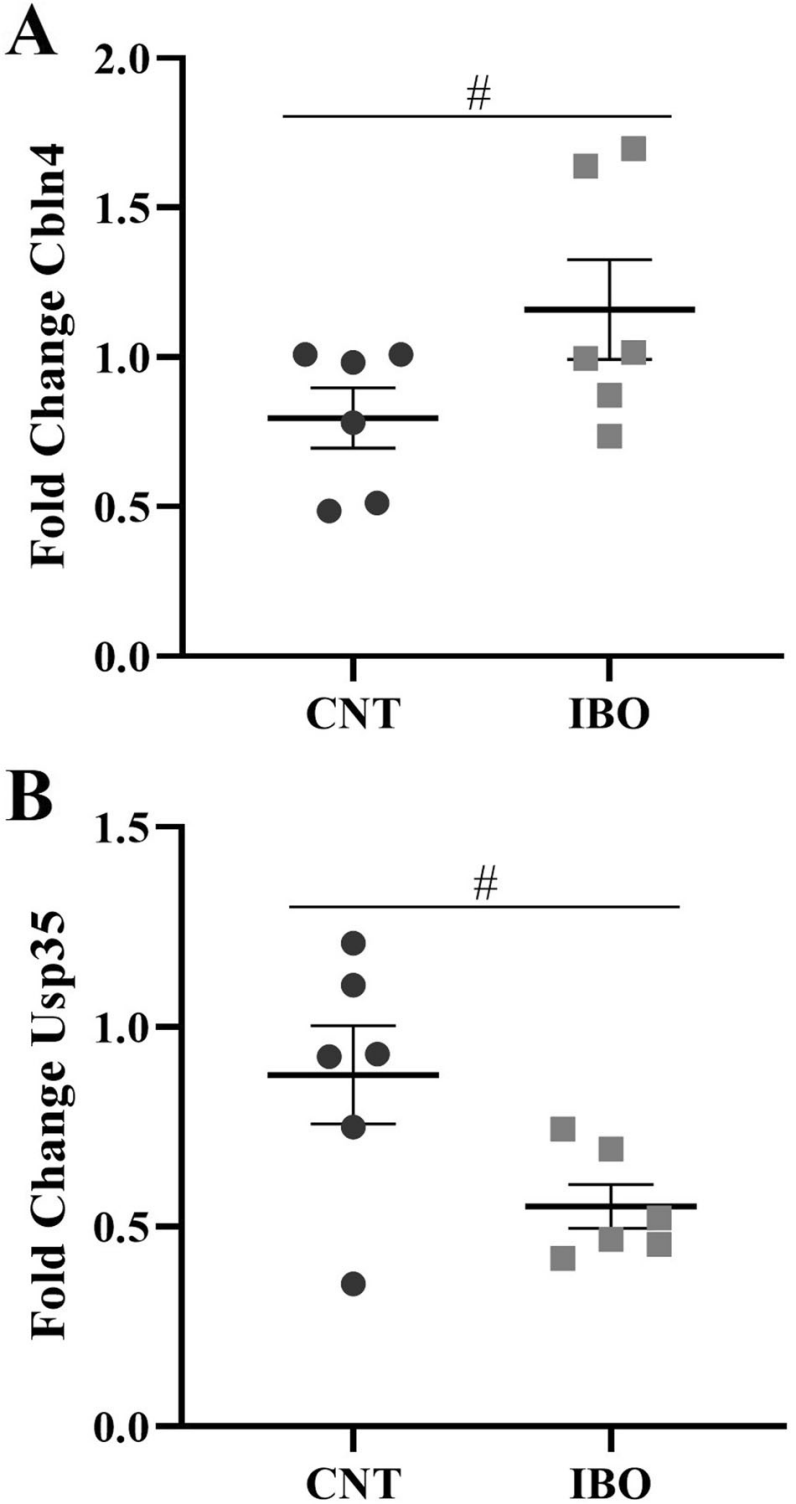
Frontal cortex gene expression determined by qPCR with treatment differences. *Cbln4* (**A**) and *Usp35* (**B**). The symbol # indicates differences between treatments at *p* < 0.05.

A two-way ANOVA (sex and treatment) analysis of the variance showed significant effects of sex in both *Cbln4* and *Cbln2* genes (*Cbln4* [*F*_1,11_ = 15.777, *p* = 0.004] and *Cbln2* [*F*_1,11_ = 8.904, *p* = 0.017]). Females showed less expression compared to males in both cases (Fig. 1C, D). Treatment effects were only observed on two genes: *Cbln4* ([*F*_1,11_ = 8.483, *p* = 0.020]) (Fig. 2A) showed an upregulation in IBO-treated subjects and *Usp35* ([*F*_1,11_ = 6.698, *p* = 0.032]) (Fig. 2B) showed a downregulation in IBO-treated subjects. No significant effects were observed in the rest of the analyzed genes (Fig. 1). It is important to mention that two outliers, one in males and one in females, were excluded from the statistical analyses for the group of IBO-treated mice in relation to *Oxt* and *Avp* expression. These outliers exhibited significantly higher expression levels, hundreds of times greater than the mean expression level of the group.

## Discussion

This study aimed to identify changes in gene expression in the frontal cortex in mice 4 h after a single oral dose of IBO. To date, this is the first time that transcriptomics has been used to study IBO’s mechanisms of action. Following the transcriptomic analysis, it was observed that genes associated with hormonal pathways and synaptogenesis were upregulated by IBO. Conversely, genes involved in apoptotic processes and endosomal transports showed downregulation. Validation of gene expression through qPCR confirmed the observed results, except for the genes related to hormonal pathways.

Due to the limited understanding of IBO’s mechanisms of action, there is a significant need to use new techniques to identify new targets and potential mechanisms of action and since transcriptomic is an exploratory approach by nature, there was a high degree of variability among different samples. However, certain patterns can be observed. First, a general difference in sex was clearly observed. Males showed changes in eight genes when comparing CNT and IBO conditions, whereas females had modifications in 28 genes. This might be attributed to IBO’s greater bioavailability in females, as reported in preclinical studies [33, 34]. However, further research should confirm these findings persist for longer periods to explain the long-term effects of IBO.

Both *Oxt* and *Avp* genes were upregulated, indicating their involvement in hormonal pathways as they encode oxytocin and vasopressin, respectively. While the potential involvement of the neuroendocrine system in therapeutic outcomes has been explored in the context of other psychedelics [38, 54–56], there is currently no evidence of this in relation to IBO. In contrast with classic psychedelics, the potential effect of IBO in the neuroendocrine system would not be mediated by the stimulation of 5-HT2A receptors, as it does not bind to that receptor. Indeed, the only study assessing the neuroendocrine effects of IBO found an absence of effect on cortisol levels [57]. Studies have reported that LSD [37, 38], MDMA [58], and mescaline [37] can increase oxytocin levels. This increase in oxytocin may be directly associated with the prosocial effects [59–61] and promotion of neuroplasticity [37] observed with psychedelics. In a recent study, IBO was shown to reinstate social reward learning for more than 4 weeks after an acute administration [62], so a putative mechanism could be the promoting effect of *Oxt*.

Vasopressin has also been associated with prosocial effects [59, 63, 64]. Additionally, low levels of this hormone have been associated with depression or psychotic disorders [65, 66]. In regards to substance use disorders, it has been observed that the central administration of vasopressin blocks amphetamine-induced conditioned place preference in rats [67]. It is believed that the septum/vasopressin system modulates the release of neurotransmitters in the reward system [68]. IBO’s possible modulation of both *Oxt* and *Avp* may have direct implications for understanding its anti-addictive effects. This is particularly relevant given the recent advancements in our understanding of the roles oxytocin and vasopressin may have in substance use disorders [64]. However, the validation analysis with qPCR could not confirm the overexpression of *Oxt* or *Avp* in the obtained samples. This might be due to the high variability found between subjects and differences between sexes. Based on these, future studies should include brain areas such as the hypothalamus where the expression of *Oxt* [69] or *Avp* [67] is high.

*Cbln2* and *Cbln4*, cerebellins belonging to the C1q and tumor necrosis factor, have a strong association with synaptogenesis [70, 71] and also showed increased expression in transcriptomic analysis. Their upregulation suggests that IBO is inducing cellular-level neuroplasticity. To date, the main mechanism by which IBO induces neuroplasticity has been restricted to both glial- and brain-derived neurotrophic factors [32, 72]. However, a prior study has reported that it is actually NOR, the principal metabolite of IBO, and not IBO itself that induces neuroplasticity [73]. It is worth noting that a significant increase in these cerebellins, particularly *Cbln4*, was observed following IBO administration in males but not females. Future studies using larger samples should explore these potential differences by sex in depth. The overexpression of cerebellins reported in transcriptomic analysis was confirmed by qPCR. Furthermore, there was an observed increase in the *Nptx2* gene, which was also related to the synaptogenesis of excitatory neurons and related to AMPA receptor synapse clustering in IBO-treated females.

The ubiquitin-specific peptidase 35 adaptor (*Usp35*) gene was downregulated after IBO administration in both males and females. This gene is associated with apoptotic processes, although its specific role is not yet clear. While some studies suggest that *Usp35* is a tumor suppressor [51, 74], others point to an upregulation of *Usp35* in ovarian cancer [75]. Four different isoforms of *Usp35* have been identified so far [76]. It is therefore possible that different pathways modulating specific isoforms lead to distinct effects. The downexpression of *Usp35* reported in transcriptomics analysis was confirmed by qPCR. Nevertheless, other genes related to apoptotic processes or cell growth were also found to be upregulated by IBO in females (*Tnfrsf25*, *Cdkn1a*, and *Phlad1*) [77, 78], while those negatively regulating these processes were downregulated (*Ccdc154* and *Dhr2*) [79, 80].

The gene *Ap5b1* was also downregulated in the IBO group. This gene is associated with endosomal transport. It is challenging to suggest specific implications of this gene’s downregulation. Similarly, there were several other genes affected by IBO for which specific functions are not yet known because they were predicted or uncharacterized (e.g., *Gm34306*, *Gm51898*, *Gm44505*, among others), or are related to complex systems such as the immune and inflammatory system (e.g., *Il1rn*, *Eomes*, *Sh2d1b1*, *Cd19*, *Egr4*) or calcium ion channels (*Smim3*, *Slc25a25*, *Mcu*). These effects on the immune and inflammatory systems open new therapeutic implications.

The main limitation of this study was the high variability observed in the transcriptomic analyses which highlights the need for a larger sample, especially to better explore differences observed between sexes. Additionally, another limitation was the collection of only one measurement at +4 h post-IBO administration. Future studies should investigate changes in gene expression at various time points, including long-term assessments to better understand IBO’s sustained effects. This approach is crucial given the numerous reports of long-term behavioral changes in observational research. While the long-lasting action of NOR, ibogaine’s metabolite, has been suggested as the potential cause of these effects [81], it is essential to consider the possibility of gene expression modifications by both IBO and NOR. Further investigations should address these limitations and explore the complex interplay between gene expression changes and the behavioral outcomes of IBO and its metabolite NOR.

In conclusion, this study represents a significant step forward in understanding the molecular mechanisms underlying the effects of IBO. Through the application of transcriptomic analysis, we have identified notable changes in gene expression following a single dose of IBO in mice. Our findings reveal that the genes involved in hormonal pathways and synaptogenesis were upregulated by IBO. Conversely, the genes associated with apoptotic processes and endosomal transports were downregulated. These results were further validated through quantitative polymerase chain reaction (qPCR). It’s important to note that the validation of gene expression pertaining to hormonal pathways did not completely corroborate the findings of the transcriptomic analysis. In addition, we also observed general sex differences, with females showing more alterations in gene expression after IBO treatment. Overall, this study expands our knowledge of IBO’s molecular actions and underscores the potential of omics techniques in investigating the effects of psychedelic drugs. Further research is warranted to study the contribution of each of the identified genes at different time points to establish acute and long-term effects after IBO treatment, specifically for those pathways involved in neuromodulation. The precise mechanisms through which IBO modulates gene expression are especially relevant to identifying new therapeutic applications.

## Data Availability

The data that support the findings of this study and the R script analyses are available from the corresponding author upon reasonable request. Read counts of RNA sequencing are available at 10.34810/data912.
